# Supplementary figures and images for: Oculopharyngeal muscular dystrophy (OPMD) associated alanine expansion impairs the function of the nuclear polyadenosine RNA binding protein PABPN1 as revealed by proximity labeling and comparative proteomics
Source: PLoS Genet. 2026 Jan 26;22(1):e1011743. doi: 10.1371/journal.pgen.1011743 (PMC12858073; doi:10.1371/journal.pgen.1011743)

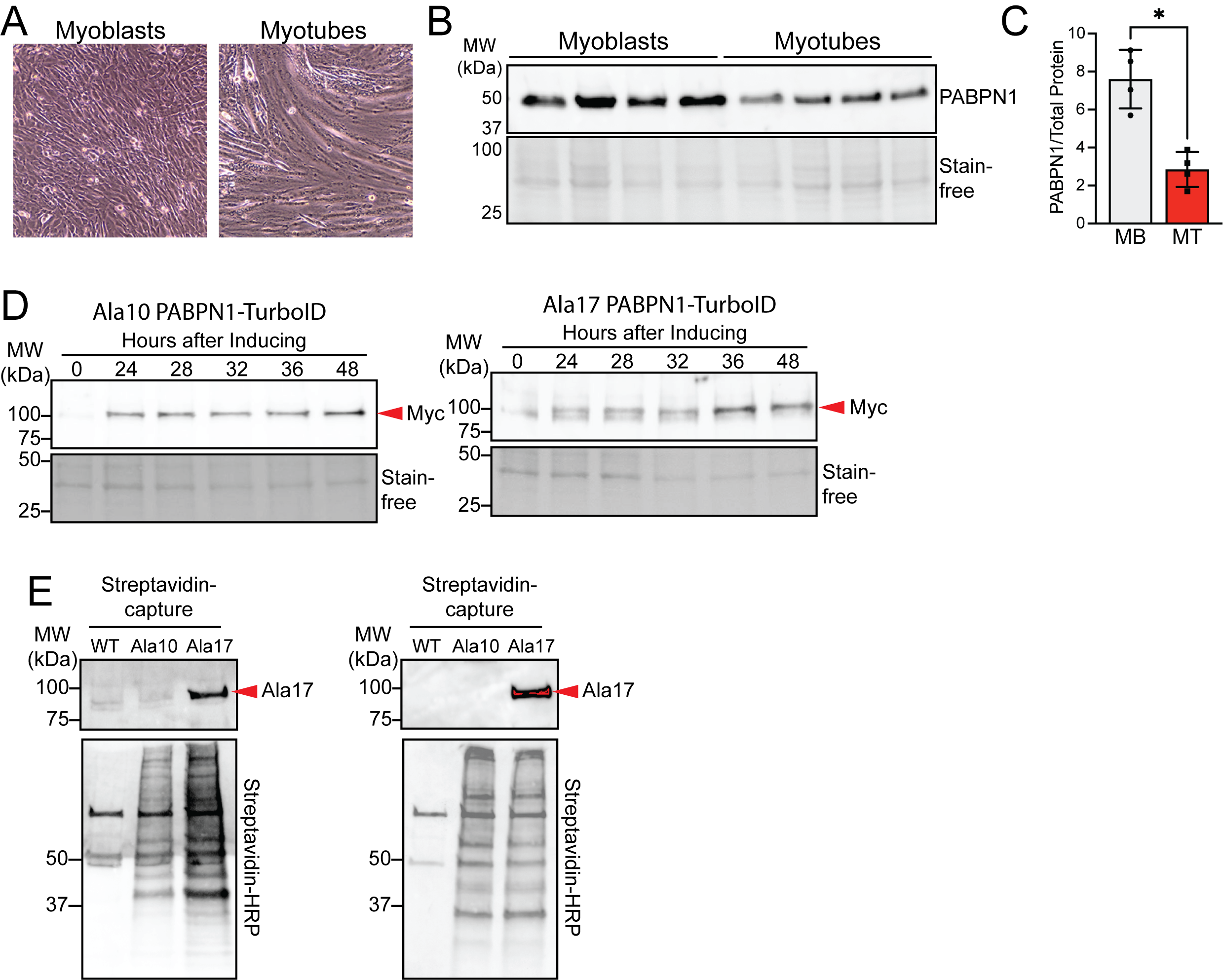

Supplement: S1 Fig — A) Representative phase contrast image of C2C12 myoblasts versus myotubes. B) Immunoblot probed with an antibody to PABPN1 showing decreased PABPN1 in myotubes compared to myoblasts. C) Quantification of immunoblot in B. Shown is mean ± standard deviation for n = 4 experiments. Statistical significance determined using paired t-test. * p < 0.05. D) Immunoblots showing time course of doxycycline-induced expression of Ala10 (left) and Ala17 (right) PABPN1-TurboID constructs as detected using an antibody to the Myc tag. Stain-free imaging used as a loading control. E) Additional replicates of n2 (left) and n3 (right) blots of streptavidin elutions from Ala10 and Ala17 PABPN1-TurboID expressing myotubes probed with an antibody to the alanine expansion (top) to show alanine expansion in Ala17 PABPN-TurboID or probed with streptavidin-HRP to show increased presence of biotinylated proteins compared to wild type (WT) control myotubes. (TIF) [file pgen.1011743.s001.tif]

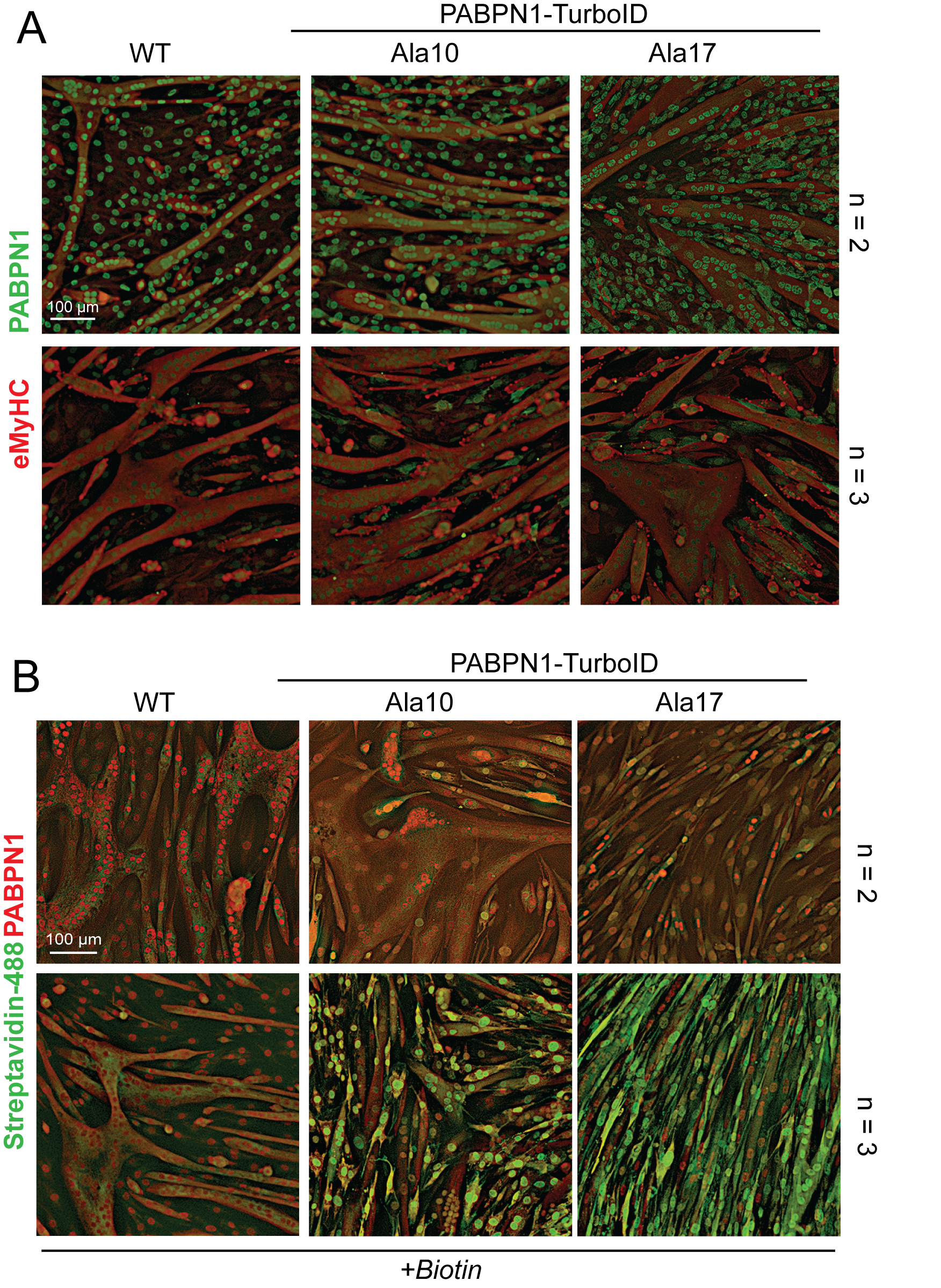

Supplement: S2 Fig — A) Additional immunostaining of n2 (top) and n3 (bottom) with an antibody to PABPN1 (green) in myotubes from WT and stable cells expressing near-native levels of Ala10 and Ala17 PABPN1-TurboID. Immunostaining with an antibody to embryonic myosin heavy chain (red) was used to detect myotubes. B) Additional staining of n2 (top) and n3 (bottom) using AF488-conjugated streptavidin (green) to detect biotin and an antibody to PABPN1 (red). (TIF) [file pgen.1011743.s002.tif]

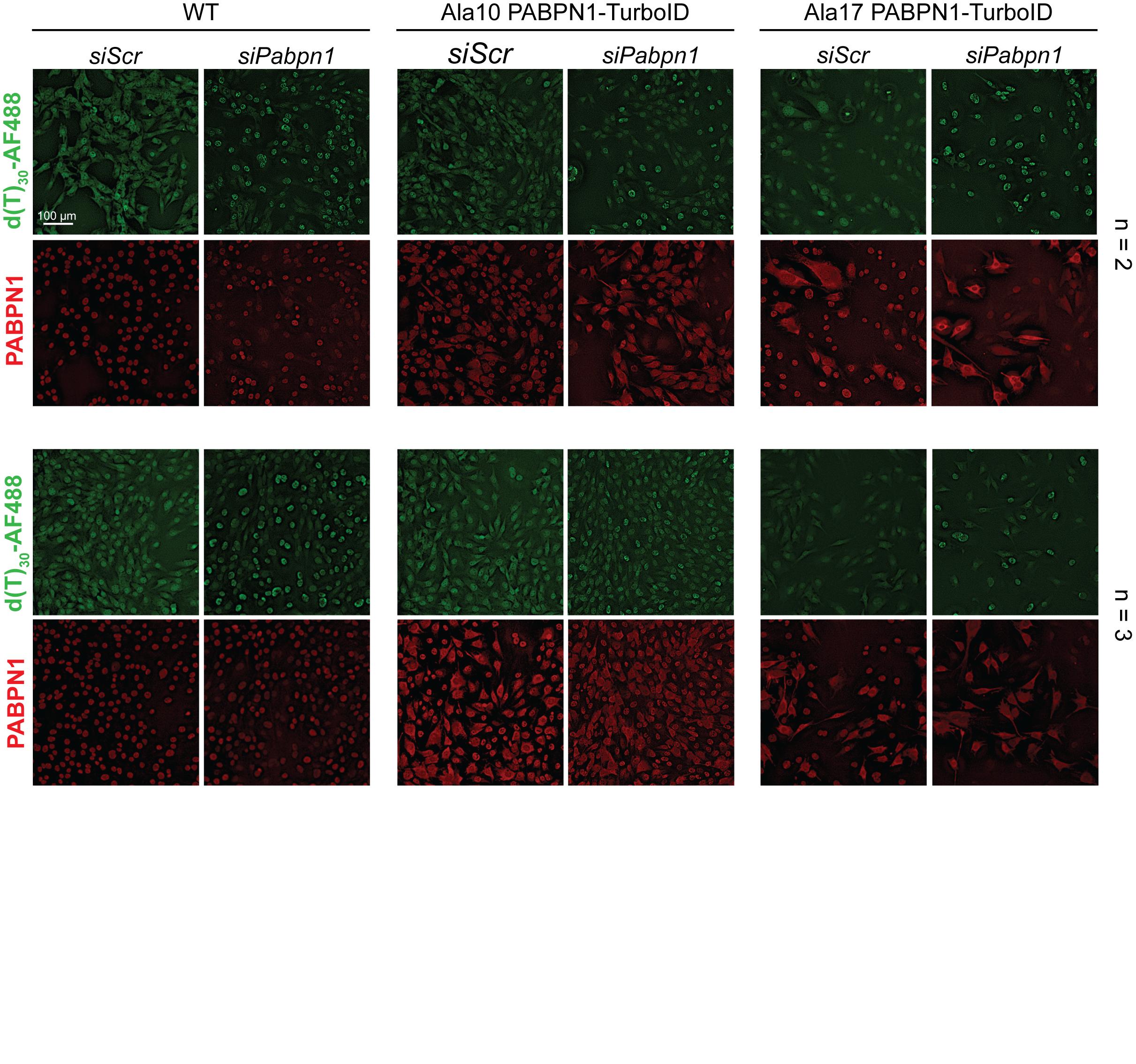

Supplement: S3 Fig — Additional d(T)30-AF488 FISH staining of n2 (top) and n3 (bottom) in Pabpn1 knockdown in WT myoblasts or myoblasts expressing Ala10 or Ala17 PABPN1-TurboID. (TIF) [file pgen.1011743.s003.tif]

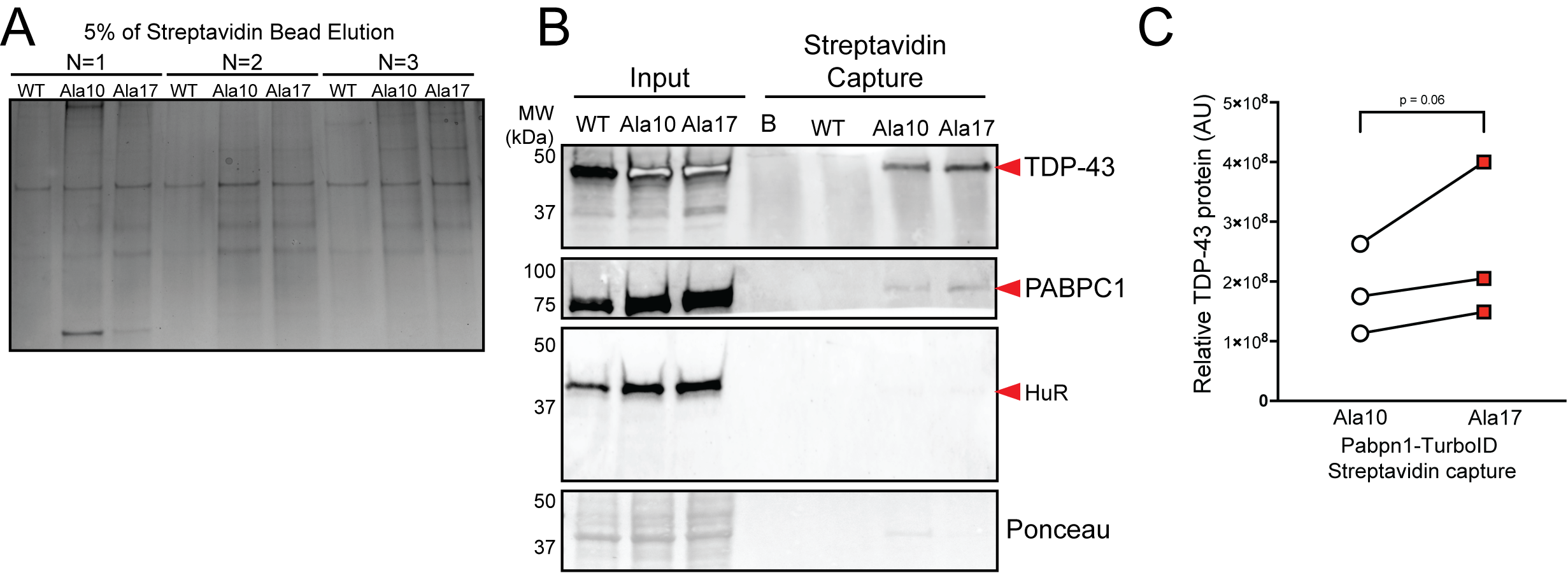

Supplement: S4 Fig — A) Silver-stained gel of 5% of streptavidin bead elutions from WT myotubes and myotubes expressing Ala10 or Ala17 PABPN1-TurboID. Shown are all three replicates used for comparative proteomics. B) Immunoblot of streptavidin elutions from WT myotubes and myotubes expressing Ala10 or Ala17 PABPN1-TurboID probed with antibodies to known PABPN1 binding partners TDP-43 and PABPC1 as well as HuR. Ponceau stain is used as a loading control. C) Quantification of TDP-43 band from streptavidin elution blot showing trending increase in TDP-43 detection in Ala17 PABPN1-TurboID proximal blot. Shown are n = 3 replicates analyzed by paired t test. (TIF) [file pgen.1011743.s004.tif]

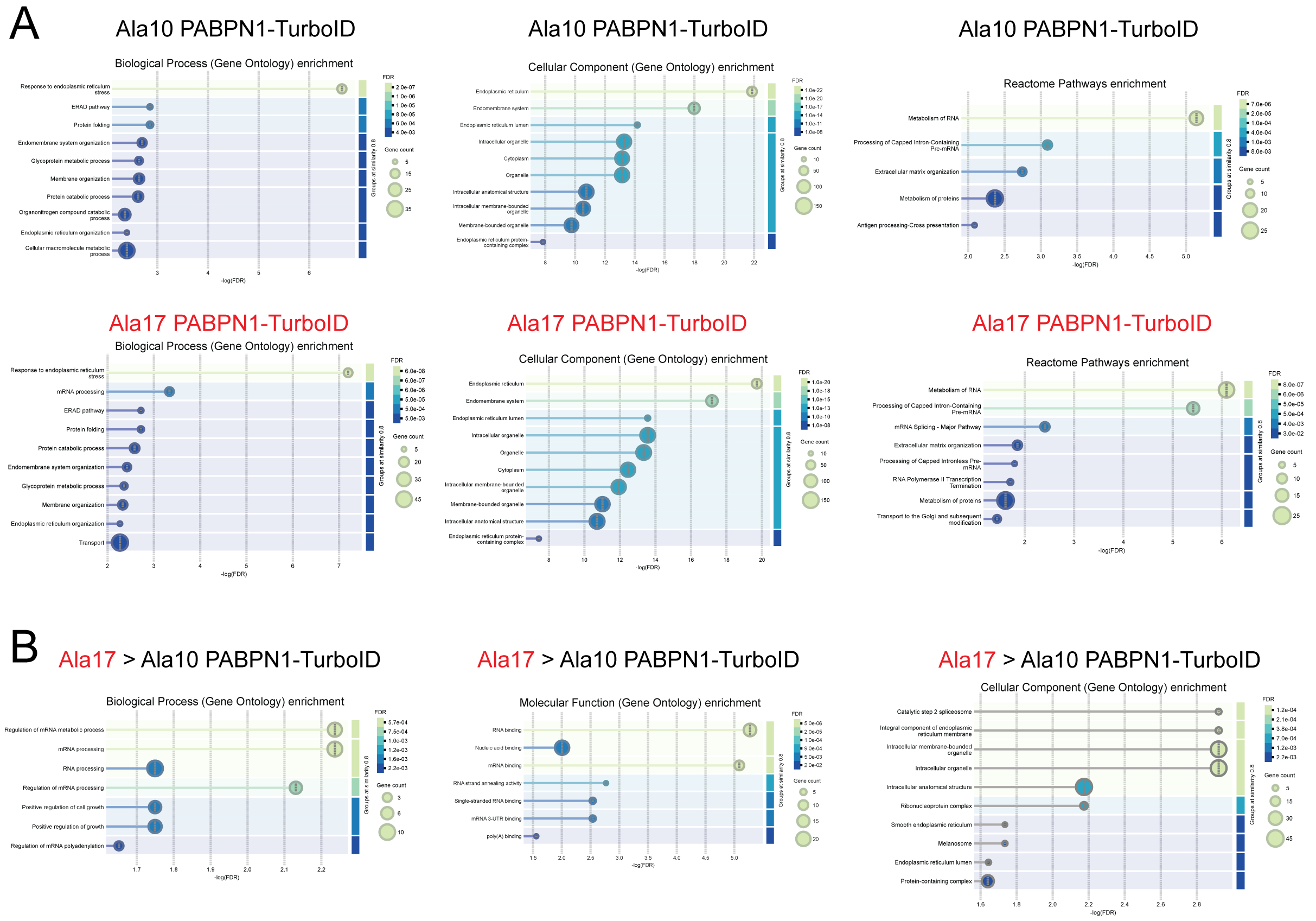

Supplement: S5 Fig — A) Gene ontology- biological process, cellular component, and Reactome pathways enriched in Ala10 PABPN1-TurboID (top) and Ala17 PABPN1-TurboID (bottom) proximal proteins. B) Gene ontology- biological process, cellular component, and Reactome pathways enriched in Ala17 PABPN1-TurboID proximal proteins compared to Ala10 PABPN1-TurboID. In all cases, data shown are representative of proteins detected in at least two of three Ala10 or Ala17 PABPN1-TurboID replicates and not detected in WT controls. (TIF) [file pgen.1011743.s005.tif]

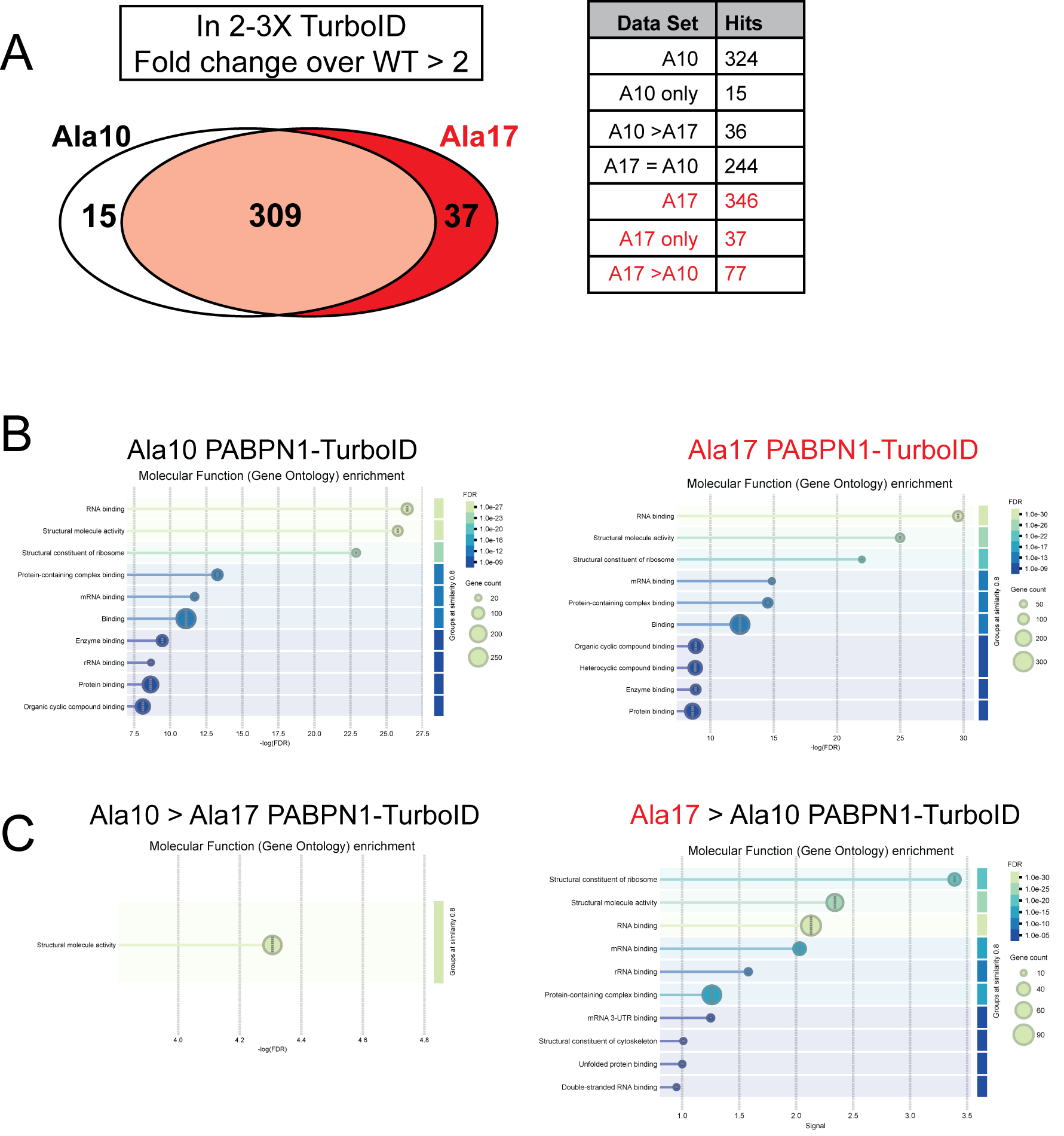

Supplement: S6 Fig — A) Proximal proteins detected in at least two of three PABPN1-TurboID replicates at least two-fold higher or more over WT controls. B) Gene ontology-molecular function enrichment for Ala10 PABPN1-TurboID (left) and Ala17 PABPN1-TurboID (right) proximal proteins. C) Gene-ontology molecular function enrichment for proteins enriched in Ala10 over Ala17 PABPN1-TurboID (left) and Ala17 over Ala10 PABPN1-TurboID (right). (TIF) [file pgen.1011743.s006.tif]
