# Supplementary material for: Oculopharyngeal muscular dystrophy (OPMD) associated alanine expansion impairs the function of the nuclear polyadenosine RNA binding protein PABPN1 as revealed by proximity labeling and comparative proteomics
Source: PLoS Genet. 2026 Jan 26;22(1):e1011743. doi: 10.1371/journal.pgen.1011743 (PMC12858073; doi:10.1371/journal.pgen.1011743)
Supplement: S7 Fig — (S7_Fig.PDF) [file pgen.1011743.s007.pdf]

SUPPLEMENTAL FIGURE 7: NUMERICAL DATA FOR ALL GRAPHS

FIGURE 1D: Dose response curve for PABPN1-TurboID

Dose dox	A10			A17		
0.5	26.6471	9.31608	15.3687	25.8245	9.9204	8.52102
0.75	34.6971	24.0346	46.2835	32.0203	31.0699	77.9972
1	44.3702	43.1778	59.1461	61.5856	75.4121	110.201

FIGURE 2B: Cytoplasmic/nuclear ratio of PABPN1-TurboID

Cells	A10-Turbo	A17-Turbo
Cyto/nuc ratio (A))	0.235504	0.441512
	0.190951	0.159992
	0.156567	0.19082
	0.255121	0.966511
	0.264197	0.021055
	0.600129	

FIGURE 2C: Cytoplasmic/nuclear ratio of endogenous PABPN1

Cells	WT	A10-Turbo	A17-Turbo
Cyto/nuc ratio (AU)	0.049643	0.030487	0.12504
	0.03981	0.016682	0.02384
	0.034443	0.007556	0.01212
	0.006029	0.014487	0.0185
	0.000863	0.016727	0.00188
	0.00778	0.038224	0.0369

FIGURE 3B: TOTAL NUCLEI IN PABPN1 KNOCKDOWN MYOBLASTS WITH PABPN1 TURBOID RESCUE

	WT	A10-TURBO	A17-TURBO
siScr	1171	967	367
	1278	1200	755
	1434	1122	491
siPabpn1	1033	661	378
	1327	1234	1004
	1570	1625	379

FIGURE 3E: FUSION INDEX PABPN1 KNOCKDOWN WITH ALA10 PABPN1-TURBOID RESCUE

Scr	KD	KD + Ala10
17.8942	0	15.70872
36.5326	13.6627	35.2834
26.8219	6.88538	24.32486
47.1375	5.97954	52.11606

FIGURE 3H: FUSION INDEX PABPN1 KNOCKDOWN WITH ALA17 PABPN1-TURBOID RESCUE

Scr	KD	KD + Al17
43.4074	14.9853	29.05452
41.1967	1.88296	22.03776
34.9448	13.4197	18.36925
24.1608	3.9428	19.10227

FIGURE 3I: FUSION INDEX siSCR CONTROL CELLS WITH ALA10 OR ALA17 PABPN1-TURBOID

	No Plasmid							
A10-TURBO	17.8942	36.53264	26.82194	47.1375	23.5892	34.7399	28.1264	52.3322
A17-TURBO	43.4074	41.1967	34.94478	24.1608	41.3364	52.1923	29.9888	25.5417
	PABPN1-TurboID							
A10-TURBO	18.4649	28.40847	25.38023	47.1613	19.7524	32.1671	35.9158	58.1395
A17-TURBO	38.5957	20.3688	19.9528	15.8646	45.1538	22.8737	29.8386	25.7065

FIGURE 3J: TOTAL NUCLEI IN DIFFERENTIATED MYOTUBES FROM CONTROL OR PABPN1 KNOCKDOWN WITH A10 OR A17 PAB TURBOID

Scr	KD	KD + Ala10	KD + Ala17
5633	854	3070	4129
4236	1082	4106	3389
4626	3279	5487	6597
1331	2469	5073	6416
3965	2542	4584	
2634	1394	3707	
4686	1223	4468	
4966	1049	4998	
7196	2369	11923	

FIGURE 5D: QUANTIFICATION OF BLOTS FROM CYCLOHEXAMIDE CHASE - PABPN1 IN WT CELLS (endogenous), A10 PABPN1-TURBO (Turbo), AND A17 PABPN1-TURBO (Turbo)

	Relative protein (AU)				
Time after CHX (h)	WT PABPN1				
0	100	100	100		
1	87.0458	86.9261	91.81699		
2	102.166	43.5021	168.2412		
4	80.584	61.9353	120.6503		
6	84.8414	37.0978	93.24873		
8	66.8757	79.2911	40.62512		
12	87.1116	89.2171	89.02105		
24	65.3452	36.475	56.67925		
Time after CHX (h)	Ala10 PABPN1-TurboID				
0	100	100	100	100	100
1	93.7633	234.908	183.5155	69.8522	136.146
2	116.847	107.337	89.19156	37.4523	159.399
4	76.7522	164.145	159.162	54.0205	139.822
6	71.1456	81.6451	180.9315	71.7854	111.998
8	86.7721	103.369	125.0269	34.1188	25.582
12		14.9867	161.3102	5.11152	22.7226
24		76.4777	74.14468	1.70722	19.9049
Time after CHX (h)	Ala17 PABPN1-TurboID				
0	100	100	100	100	100
1	127.759	74.7183	67.65138	123.511	81.1407
2	31.5084	44.9173	20.7164	35.6379	64.9087
4	21.4293	40.8372	53.72632	18.0192	65.0494
6	21.0505	30.8883	35.90577	45.5022	54.6928
8	9.17116	21.473	50.30909	60.5896	44.1216
12		4.83608	60.78207	7.80304	26.657
24		0.04978	27.74876	0.10798	11.5038

FIGURE 5E: QUANTIFICATION OF BLOTS FROM CYCLOHEXAMIDE CHASE - ENDOOGENOUS PABPN1 IN CELLS EXPRESSING ALA10 OR ALA17 PABPN1-TURBOID

	Relative protein (AU)					
Time after CHX (h)	PABPN1 IN A10 PAB TURBO			PABPN1 IN A17 PAB TURBO		
0	100	100	100	100	100	100
1	65.946	156.201	116.3829	67.4262	98.9668	95.24
2	124.232	152.497	57.16773	55.6907	71.8684	66.1859
4	108.542	133.66	131.8787	88.0398	58.9851	72.7931
6	66.4316	95.7866	116.9471	65.1214	50.7533	43.792
8	84.8276	142.866	179.0098	3.0736	50.8771	45.941
12		71.9708	289.5851		30.9838	47.8278
24		116.234	157.502		25.9249	33.4724

FIGURE 6 B: QUANTIFICATION OF RELATIVE NUCLEAR EXPORT PROTEIN LEVELS IN MYOBLASTS VERSUS MYOTUBES

	**Relative levels (AU)**			
PROTEIN	MYOBLASTS			
ALYREF	4.9E+08	4.2E+08	4.5E+08	5.1E+08
THOC1	3.9E+08	2.2E+08	2.8E+08	2.8E+08
THOC2	1.2E+08	4.5E+07	5.4E+07	3.8E+07
THOC5	3.7E+08	2.7E+08	3.1E+08	3.5E+08
ZC3H11A	3.3E+08	2.5E+08	2.8E+08	2.7E+08
PABPN1	4E+08	2.2E+08	3.4E+08	3.3E+08
	**Relative levels (AU)**			
PROTEIN	MYOTUBES			
ALYREF	3.1E+08	3.5E+08	4.3E+08	3.8E+08
THOC1	1.3E+08	1.7E+08	2E+08	1.2E+08
THOC2	2.2E+07	3.4E+07	6E+07	2.7E+07
THOC5	2.1E+08	2.3E+08	2.4E+08	1.6E+08
ZC3H11A	2.3E+08	2.5E+08	2.8E+08	2.2E+08
PABPN1	2E+08	2E+08	2.4E+08	2.2E+08

FIGURE 6D: NUCLEAR EXPORT PROTEINS IN STREPTAVIDIN CAPTURE FROM ALA10 VERSUS ALA17 PABPN1-TURBOID

	Relative levels (AU)		
PROTEIN	Ala10 PABPN1-TurboID		
THOC5	426713	127475	881804
ALYREF	165548	10243.7	14453.2
	Relative levels (AU)		
PROTEIN	Ala17 PABPN1-TurboID		
THOC5	208822	103572	728715
ALYREF	145640	7587.48	12325.5

FIGURE 6F: QUANTIFICATION OF PABPN1-TURBOID IN THOC5 REVERSE IP IN CELLS EXPRESSING ALA10 VS ALA17 PABPN1-TURBOID

Relative levels (AU)	
Ala10 PD	Ala17 PD
178497	104350
434483	199091
392771	390328

FIGURE 7 C: QUANTIFICATION OF CORRELATION COEFFICIENT - GREEN OLIGO d(T) AF-488 AND BLUE DAPI SIGNAL IN WT CELLS AND CELLS EXPRESSING NEAR NATIVE LEVELS OF PABPN1-TURBOID

WT	Ala10 NN	Ala17 NN
0.39313	0.452544829	0.570679041
0.25444	0.415091	0.482689
0.46403	0.420568	0.597908
0.51222	0.475525	0.543579
0.59003	0.567627	0.634487
0.60794	0.686006	0.693501

FIGURE 7 D: QUANTIFICATION OF CORRELATION COEFFICIENT - GREEN OLIGO d(T) AF-488 AND BLUE DAPI SIGNAL IN WT CELLS AND CELLS OVEREXPRESSING PABPN1-TURBOID

WT	Ala10 Over	Ala17 Over
0.39313	0.468653686	0.59755206
0.25444	0.412586	0.514146
0.46403	0.534368	0.590027
0.51222	0.501254	0.581196
0.59003	0.592042	0.631789
0.60794	0.68338	0.696385

FIGURE 7E: QUANTITATIVE PCR DATA SHOWING NUCLEAR/CYTOPLASMIC RATIO OF *Tnnt3* TRANSCRIPT IN WT CELLS AND CELLS EXPRESSING NEAR NATIVE LEVELS OF PABPN1-TURBOID

WT	Ala10 PABPN1-TurboID	Ala17 PABPN1-TurboID
3.305	3.684	4.06
1.49787	1.656891791	2.028595342
4.37936	3.949318229	5.18312509

FIGURE S1C: LEVELS OF PABPN1 PROTEIN IN C2C12 MYOBLASTS VERSUS MYOTUBES

Relative protein (AU)	
MB	MT
9.12	3.17925
7.04662	1.70815
8.54	2.62368
5.69445	3.8834

FIGURE S4C: RELATIVE LEVELS OF TDP-43 PROTEIN IN STREPTAVIDIN CAPTURE FROM CELLS EXPRESSING PABPN1-TURBOID

Relative protein (AU)	
A10	A17
1.8E+08	2.1E+08
1.1E+08	1.5E+08
2.6E+08	4E+08
