# Supplementary material for: Oculopharyngeal muscular dystrophy (OPMD) associated alanine expansion impairs the function of the nuclear polyadenosine RNA binding protein PABPN1 as revealed by proximity labeling and comparative proteomics
Source: PLoS Genet. 2026 Jan 26;22(1):e1011743. doi: 10.1371/journal.pgen.1011743 (PMC12858073; doi:10.1371/journal.pgen.1011743)
Supplement: S1 Table — Table includes primary and secondary antibodies and horseradish peroxidase (HRP) or AF-488 conjugated streptavidin. (S1_Table.PDF) [file pgen.1011743.s008.pdf]

SUPPLEMENTAL TABLE 1			
**Antibody**	**Source**	**Experiment**	**Dilution/Amt**
PABPN1	Abcam ab75855	Blot	1:1000
PABPN1	Abcam ab75855	IF stain	1:250
Ala expansion	Vest, Apponi et al. 2015	Blot	1:1000
BirA	Agrisera AS20 4440	Blot	1:1000
Myc	Cell Signaling Technologies 2276	Blot	1:1000
GAPDH	Cell Signaling Technologies 5174	Blot	1:2000
Histone H3	Cell Signaling Technologies 4499	Blot	1:2000
HuR/ELAVL1	Santa Cruz sc-5261	Blot	1:1000
PABPC1	Cell Signaling Technologies 4992	Blot	1:1000
eMyHC	DSHB F1.652 hybridoma supernatant	Blot	1:100
eMyHC	DSHB F1.652 hybridoma supernatant	IF stain	1:10
ALY/REF	Santa Cruz sc-32311	Blot	1:1000
THOC5	Bethyl/Fortis A302-119	Blot	1:2000
THOC5	Abcam ab86070	IP	1 µg
TDP-43	Proteintech 10782-2-AP	Blot	1:1000
ZC3H11A	Proteintech 26081-1-AP	Blot	1:1000
HRP anti-mouse	Jackson ImmunoResearch 115-035-003	Blot	1:10,000
HRP anti-rabbit	Jackson ImmunoResearch 111-035-003	Blot	1:10,000
AF 488 anti-rabbit	Jackson ImmunoResearch 715-095-152	IF stain	1:500
AF-594 anti-rabbit	Jackson ImmunoResearch 111-035-003	IF stain	1:500
AF-488 anti-mouse	Jackson ImmunoResearch 715-585-152	IF stain	1:500
AF-594 anti-mouse	Jackson ImmunoResearch 715-585-150	IF stain	1:500
Streptavidin HRP	Cell Signaling Technologies 3999	Blot	1:10,000
Streptavidin AF-488	Invitrogen S11223	Stain	1:500
